# Supplementary material for: Modeling the mutation and reversal of engineered underdominance gene drives
Source: J Theor Biol. 2019 Oct 21;479:14–21. doi: 10.1016/j.jtbi.2019.06.024 (PMC6699728; doi:10.1016/j.jtbi.2019.06.024)
Supplement: Supplementary Data S1 — Supplementary Raw Research Data. This is open data under the CC BY license http://creativecommons.org/licenses/by/4.0/ [file mmc1.pdf]

Supplementary Information: Modeling the mutation and reversal of engineered underdominance gene drives

M.P. Edgington and L.S. Alphey
The Pirbright Institute, Ash Road, Woking, Surrey, GU24 0NF, UK.

1 Mathematical Stability Analysis

Here we outline the workings of mathematical stability analyses intended to complement the results given in the main text. In particular, we begin by analysing the stability of equilibria resulting from a deterministic population genetics model of a two-locus underdominance gene drive system in absence of any free-suppressor transgenic constructs. This model is then extended to include free-suppressor transgenic constructs and the resulting equilibria re-analysed to explore the implications of releasing free-suppressor transgenic constructs.

1.1 Model 1: Two-Locus Engineered Underdominance

We begin here by outlining a simple haplotype-based population genetics model of a two-locus engineered underdominance system in absence of any resistance or free-suppressor constructs. Here we denote haplotypes by a pair of letters where upper case (A or B) denotes the presence of a transgenic construct and lower case (a or b) denotes wild type. This gives a total of four possible haplotypes AB, Ab, aB and ab, however upon assuming that fitness costs associated with each transgenic construct are equal we see that Ab and aB frequencies are equal. We therefore adjust the model using the relationship $Ab = aB$ to yield the following set of three difference equations.

$$AB_{t+1} = \left[\varepsilon^4 AB_t^2 + 2\varepsilon^3 AB_t Ab_t + \frac{1}{2}\varepsilon^2 AB_t ab_t + \frac{1}{2}\varepsilon^2 Ab_t^2\right] / \Omega_t \qquad = f_1 = g_1/\Omega_t \tag{1}$$

$$Ab_{t+1} = \left[\varepsilon^3 AB_t Ab_t + \frac{1}{2}\varepsilon^2 AB_t ab_t + \frac{1}{2}\varepsilon^2 Ab_t^2\right] / \Omega_t \qquad = f_2 = g_2/\Omega_t \tag{2}$$

$$ab_{t+1} = \left[\frac{1}{2}\varepsilon^2 AB_t * ab_t + \frac{1}{2}\varepsilon^2 Ab_t^2 + ab_t^2\right] / \Omega_t \qquad = f_3 = g_3/\Omega_t \tag{3}$$

Within these equations ε represents the relative fitness of an individual carrying a transgenic construct. These are applied multiplicatively such that individuals carrying n transgenic constructs have a relative fitness of ε^n. In each equation Ω is given by

$$\Omega_t = \varepsilon^4 AB_t^2 + \varepsilon^3 4AB_t Ab_t + \varepsilon^2 \left(2Ab_t^2 + 2AB_t ab_t\right) + ab_t^2, \tag{4}$$

and denotes the overall fitness of a population relative to a fully wild-type equivalent.

In order to conduct a stability analysis on the equilibria resulting from this model it is necessary to derive the Jacobian matrix (J) containing the partial derivatives of f_1, f_2 and f_3 with respect to each of the model variables (i.e. AB, Ab and ab). This Jacobian matrix is of the form

$$J = \begin{pmatrix} \frac{\partial f_1}{\partial AB} & \frac{\partial f_1}{\partial Ab} & \frac{\partial f_1}{\partial ab} \\ \frac{\partial f_2}{\partial AB} & \frac{\partial f_2}{\partial Ab} & \frac{\partial f_2}{\partial Ab} \\ \frac{\partial f_3}{\partial AB} & \frac{\partial f_3}{\partial Ab} & \frac{\partial f_3}{\partial ab} \end{pmatrix}, \tag{5}$$

with partial derivatives obtained via the quotient rule and of the form

$$\frac{\partial f_1}{\partial AB} = \left(\frac{\partial g_1}{\partial AB}\Omega - g_1\frac{\partial \Omega}{\partial AB}\right)/\Omega^2, \qquad \frac{\partial f_1}{\partial Ab} = \left(\frac{\partial g_1}{\partial Ab}\Omega - g_1\frac{\partial \Omega}{\partial Ab}\right)/\Omega^2,$$
$$\frac{\partial f_1}{\partial ab} = \left(\frac{\partial g_1}{\partial ab}\Omega - g_1\frac{\partial \Omega}{\partial ab}\right)/\Omega^2, \qquad \frac{\partial f_2}{\partial AB} = \left(\frac{\partial g_2}{\partial AB}\Omega - g_2\frac{\partial \Omega}{\partial AB}\right)/\Omega^2,$$
$$\frac{\partial f_2}{\partial Ab} = \left(\frac{\partial g_2}{\partial Ab}\Omega - g_2\frac{\partial \Omega}{\partial Ab}\right)/\Omega^2, \qquad \frac{\partial f_2}{\partial ab} = \left(\frac{\partial g_2}{\partial ab}\Omega - g_2\frac{\partial \Omega}{\partial ab}\right)/\Omega^2,$$
$$\frac{\partial f_3}{\partial AB} = \left(\frac{\partial g_3}{\partial AB}\Omega - g_3\frac{\partial \Omega}{\partial AB}\right)/\Omega^2, \qquad \frac{\partial f_3}{\partial Ab} = \left(\frac{\partial g_3}{\partial Ab}\Omega - g_3\frac{\partial \Omega}{\partial Ab}\right)/\Omega^2,$$
$$\frac{\partial f_3}{\partial ab} = \left(\frac{\partial g_3}{\partial ab}\Omega - g_3\frac{\partial \Omega}{\partial ab}\right)/\Omega^2.$$

Partial derivatives utilised in the above equations are of the following form

$$\frac{\partial \Omega}{\partial AB} = 2\varepsilon^4 AB^* + 4\varepsilon^3 Ab^* + 2\varepsilon^2 ab^*, \qquad \frac{\partial g_1}{\partial AB} = 2\varepsilon^4 AB^* + 2\varepsilon^3 Ab^* + \frac{1}{2}\varepsilon^2 ab^*,$$
$$\frac{\partial \Omega}{\partial Ab} = 4\varepsilon^3 AB^* + 4\varepsilon^2 Ab^*, \qquad \frac{\partial g_1}{\partial Ab} = 2\varepsilon^3 AB^* + \varepsilon^2 Ab^*,$$
$$\frac{\partial \Omega}{\partial ab} = 2\varepsilon^2 AB^* + 2ab^*, \qquad \frac{\partial g_1}{\partial ab} = \frac{1}{2}\varepsilon^2 AB^*,$$
$$\frac{\partial g_2}{\partial AB} = \varepsilon^3 Ab^* + \frac{1}{2}\varepsilon^2 ab^*, \qquad \frac{\partial g_3}{\partial AB} = \frac{1}{2}\varepsilon^2 ab^*,$$
$$\frac{\partial g_2}{\partial Ab} = \varepsilon^3 AB^* + \varepsilon^2 Ab^*, \qquad \frac{\partial g_3}{\partial Ab} = \varepsilon^2 Ab^*,$$
$$\frac{\partial g_2}{\partial ab} = \frac{1}{2}\varepsilon^2 AB^*, \qquad \frac{\partial g_3}{\partial ab} = \frac{1}{2}\varepsilon^2 AB^* + 2ab^*.$$

We then solve for the eigenvalues (λ) using

$$\det|J - \lambda I| = 0, \tag{6}$$

where I denotes an identity matrix with equal dimensions to J. In particular, this is solved for a given equilibrium state (AB^*, Ab^*, ab^*). For cases whereby $\max(\lambda_i) \leq 1$ an equilibrium is locally stable, whereas cases with $\max(\lambda_i) > 1$ lead to unstable equilibria.

For two-locus engineered underdominance gene drive systems there are known to be four possible equilibrium states. Solving equation (6) for each of these equilibrium states allows their associated stability characteristics to be classified as follows.

- No transgenes present (either a population without transgenic releases or one in which transgenes were eliminated) - Stable
- Fixation of transgene homozygotes (not feasible through transgenic releases into the environment) - Stable where $\varepsilon = 1$ or unstable where $\varepsilon < 1$
- Transgene introgression internal equilibrium (achieved through environmental release of transgenics) - Stable so long as $\varepsilon \geq \bar{\varepsilon}$, where $\bar{\varepsilon}$ is the threshold relative fitness for a given release ratio - equilibrium non-existent where $\varepsilon < \bar{\varepsilon}$
- Transgene introgression/elimination threshold - described in the literature as an unstable equilibrium separating transgene introgression and transgene elimination equilibria dependant on transgene release ratio and relative fitness

Eigenvalues associated with these equilibria for a range of different fitness cost scenarios are shown in Figure 1.

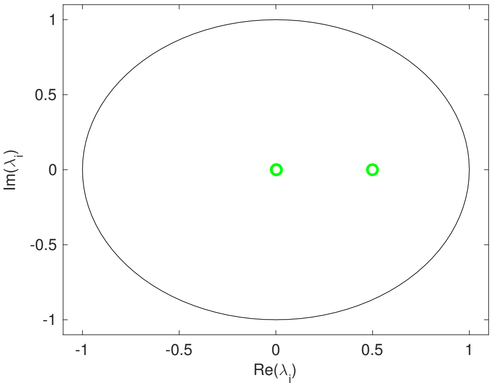

(a) Transgene Elimination ($ab = 1$)
Equivalent to Wild-Type Only

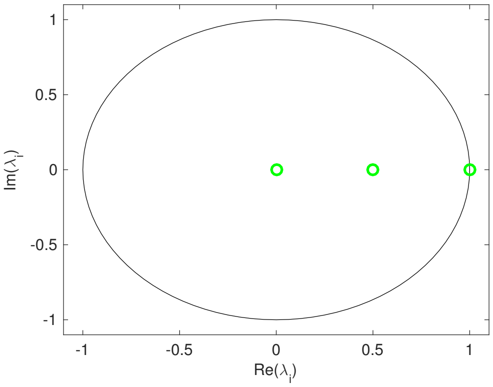

(b) Transgene Homozygous ($AB = 1$)
Zero Fitness Cost (i.e. $\varepsilon = 1$)

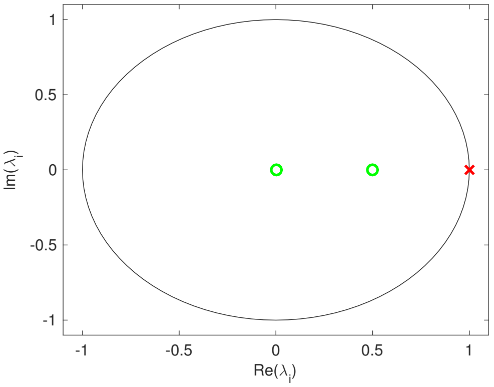

(c) Transgene Homozygous ($AB = 1$)
Tiny Fitness Cost (i.e. $\varepsilon = 0.9999$)

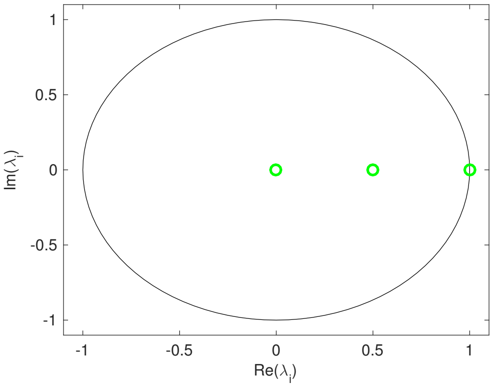

(d) Transgene Introgression ($0 < ab, Ab, AB < 1$)
Zero Fitness Cost (i.e. $\varepsilon = 1$)

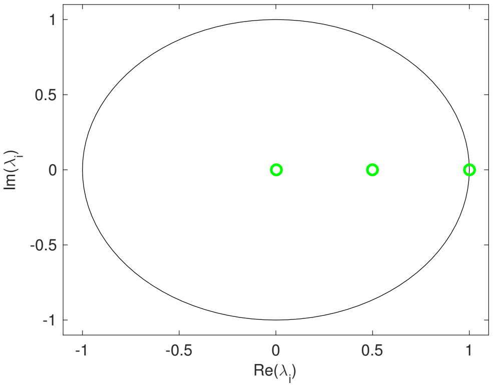

(e) Transgene Introgression ($0 < ab, Ab, AB < 1$)
Tiny Fitness Cost (i.e. $\varepsilon = 0.9999$)

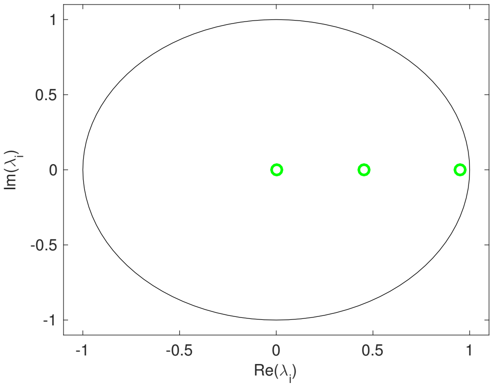

(f) Transgene Introgression ($0 < ab, Ab, AB < 1$)
5% Fitness Cost (i.e. $\varepsilon = 0.95$)

Figure 1: **Eigenvalues of various equilibrium states in absence of any free-suppressor releases.** Here green circles represent eigenvalues with absolute value less than one and red crosses eigenvalues with absolute value greater than one. Panel (a) shows that the equilibrium associated with either a fully wild-type population or the elimination of transgenes is stable. An equilibrium with only transgene homozygotes (only feasible in a specifically bred laboratory population) is found to be stable in the absence of fitness costs (panel (b)) and unstable in the presence of even a very small fitness cost (panel(c)). Panels (d)-(f) show that the stability of the internal equilibrium achieved through the release of transgenic individuals is stable in the presence of a fitness cost - so long as that fitness cost is lower than some threshold value for which transgene introgression could not be achieved by releasing individuals into the wild.

1.2 Model 2: Two-Locus Engineered Underdominance with Free-Suppressors

We now extend the previous model to include the possibility of releasing individuals carrying free-suppressor constructs (i.e. transgenic constructs possessing only the suppressor components of the original underdominance gene drive system). In this case we now obtain nine different haplotypes AB, Ab, aB, ab, A_SB_S, A_SB, AB_S, A_Sb and aB_S where the S in subscript denotes presence of a free-suppressor transgenic construct. As before we assume equal relative fitness for each transgenic construct and also equal relative fitness for each free-suppressor construct. This allows us to remove three equations from the model by noting that $Ab = aB$, $A_SB = AB_S$ and $A_Sb = aB_S$. The mathematical model is thus given by the following set of six difference equations.

$$AB_{t+1} = \Big[\varepsilon^4 AB_t^2 + 2\varepsilon^3 AB_tAb_t + \frac{1}{2}\varepsilon^2 AB_tab_t + \frac{1}{2}\varepsilon^2 Ab_t^2 + \frac{1}{2}\varepsilon^2\varepsilon_S^2 AB_tA_SB_{S_t} + 2\varepsilon^3\varepsilon_S AB_tA_SB_t \quad (7)$$
$$+\varepsilon^2\varepsilon_S AB_tA_Sb_t + \varepsilon^2\varepsilon_S Ab_tA_SB_t + \frac{1}{2}\varepsilon^2\varepsilon_S^2 A_SB_t^2\Big]/\Omega_t \quad = f_1 \quad = g_1/\Omega_t$$

$$Ab_{t+1} = \Big[\varepsilon^3 AB_tAb_t + \frac{1}{2}\varepsilon^2 AB_tab_t + \frac{1}{2}\varepsilon^2\varepsilon_S AB_tA_Sb_t + \frac{1}{2}\varepsilon^2 Ab_t^2 + \frac{1}{2}\varepsilon\varepsilon_S^2 Ab_tA_SB_{S_t} \quad (8)$$
$$+\frac{3}{2}\varepsilon^2\varepsilon_S Ab_tA_SB_t + \frac{1}{2}\varepsilon\varepsilon_S Ab_tA_Sb_t + \frac{1}{2}\varepsilon\varepsilon_S ab_tA_SB_t + \frac{1}{2}\varepsilon\varepsilon_S^2 A_SB_tA_Sb_t\Big]/\Omega_t \quad = f_2 \quad = g_2/\Omega_t$$

$$ab_{t+1} = \Big[\frac{1}{2}\varepsilon^2 AB_tab_t + \frac{1}{2}\varepsilon^2 Ab_t^2 + \varepsilon\varepsilon_S Ab_tA_Sb_t + ab_t^2 + \frac{1}{2}\varepsilon_S^2 ab_tA_SB_S + \varepsilon\varepsilon_S ab_tA_SB_t \quad (9)$$
$$+2\varepsilon_S ab_tA_Sb_t + \frac{1}{2}\varepsilon_S^2 A_Sb^2\Big]/\Omega_t \quad = f_3 \quad = g_3/\Omega_t$$

$$A_SB_{S_{t+1}} = \Big[\frac{1}{2}\varepsilon^2\varepsilon_S^2 AB_tA_SB_{S_t} + \varepsilon\varepsilon_S^2 Ab_tA_SB_{S_t} + \frac{1}{2}\varepsilon_S^2 ab_tA_SB_{S_t} + \varepsilon_S^4 A_SB_{S_t}^2 + 2\varepsilon\varepsilon_S^3 A_SB_{S_t}A_SB_t \quad (10)$$
$$+2\varepsilon_S^3 A_SB_{S_t}A_Sb_t + \frac{1}{2}\varepsilon^2\varepsilon_S^2 A_SB_t^2 + \varepsilon\varepsilon_S^2 A_SB_tA_Sb_t + \frac{1}{2}\varepsilon_S A_Sb_t^2\Big]/\Omega_t \quad = f_4 \quad = g_4/\Omega_t$$

$$A_SB_{t+1} = \Big[\frac{1}{2}\varepsilon^2\varepsilon_S^2 AB_tA_SB_{S_t} + \varepsilon^3\varepsilon_S AB_tA_SB_t + \frac{1}{2}\varepsilon^2\varepsilon_S AB_tA_Sb_t + \frac{1}{2}\varepsilon^2\varepsilon_S Ab_tA_SB_t \quad (11)$$
$$+\frac{1}{2}\varepsilon\varepsilon_S^2 Ab_tA_SB_{S_t} + \varepsilon^2\varepsilon_S Ab_tA_SB_t + \frac{1}{2}\varepsilon\varepsilon_S Ab_tA_Sb_t + \varepsilon\varepsilon_S^3 A_SB_{S_t} + \frac{3}{2}\varepsilon^2\varepsilon_S^2 A_SB_t^2$$
$$+\frac{3}{2}\varepsilon\varepsilon_S^2 A_SB_tA_Sb_t + \frac{1}{2}\varepsilon\varepsilon_S ab_tA_SB_t\Big]/\Omega_t \quad = f_5 \quad = g_5/\Omega_t$$

$$A_Sb_{t+1} = \Big[\frac{1}{2}\varepsilon^2\varepsilon_S AB_tA_Sb_t + \frac{1}{2}\varepsilon\varepsilon_S AB_tA_SB_{S_t} + \frac{1}{2}\varepsilon^2\varepsilon_S^2 Ab_tA_SB_t + \frac{1}{2}\varepsilon\varepsilon_S Ab_tA_Sb_t \quad (12)$$
$$+\frac{1}{2}\varepsilon_S^2 ab_tA_SB_{S_t} + \frac{1}{2}\varepsilon\varepsilon_S ab_tA_SB_t + \varepsilon_S ab_tA_Sb_t + \varepsilon_S^3 A_SB_{S_t}A_Sb_t + \frac{3}{2}\varepsilon\varepsilon_S A_SBA_Sb$$
$$+\varepsilon_S A_Sb_t^2 + \frac{1}{2}\varepsilon_S^2 A_Sb_t^2\Big]/\Omega_t \quad = f_6 \quad = g_6/\Omega_t$$

Here, the parameter ε retains its previous definition whereas ε_S represents the relative fitness of an individual carrying a free-suppressor transgenic construct. As before, the relative fitness of free-suppressor constructs is also applied multiplicatively. Note that here the presence of free-suppressor transgenic constructs means that Ω becomes

$$\Omega_t = \varepsilon^4 AB_t^2 + \varepsilon^3\left(4\varepsilon_S AB_tA_SB_t + 4AB_tAb_t\right) + \varepsilon^2\left[\varepsilon_S^2\left(2AB_tA_SB_{S_t} + 4A_SB_t\right) + \varepsilon_S\left(4AB_tA_Sb_t\right.\right. \quad (13)$$
$$\left.\left.+8Ab_tA_SB_t + 2Ab_t^2 + 2AB_tab_t\right)\right] + \varepsilon\left[\varepsilon_S^3 4A_SB_{S_t}A_SB_t + \varepsilon_S^2\left(4Ab_tA_SB_{S_t} + 8A_SB_tA_Sb_t\right)\right.$$
$$\left.+\varepsilon_S\left(4Ab_tA_Sb_t + 4ab_tA_SB_t\right)\right] + \varepsilon_S^4 A_SB_{S_t}^2 + 4\varepsilon_S A_SB_{S_t}A_Sb_t + \varepsilon_S^2\left(2ab_tA_SB_{S_t} + 4A_Sb_t^2\right)$$
$$+ 4\varepsilon_S ab_tA_Sb_t + ab_t^2,$$

and again represents the fitness of the overall population relative to a fully wild-type equivalent.

As before, we conduct stability analyses of equilibria resulting from this system by exploring the eigenvalues of the Jacobian matrix. Here this is of the form

$$J = \begin{pmatrix} \frac{\partial f_1}{\partial AB} & \frac{\partial f_1}{\partial Ab} & \frac{\partial f_1}{\partial ab} & \frac{\partial f_1}{\partial A_S B_S} & \frac{\partial f_1}{\partial A_S B} & \frac{\partial f_1}{\partial A_S b} \\ \frac{\partial f_2}{\partial AB} & \frac{\partial f_2}{\partial Ab} & \frac{\partial f_2}{\partial ab} & \frac{\partial f_2}{\partial A_S B_S} & \frac{\partial f_2}{\partial A_S B} & \frac{\partial f_2}{\partial A_S b} \\ \frac{\partial f_3}{\partial AB} & \frac{\partial f_3}{\partial Ab} & \frac{\partial f_3}{\partial ab} & \frac{\partial f_3}{\partial A_S B_S} & \frac{\partial f_3}{\partial A_S B} & \frac{\partial f_3}{\partial A_S b} \\ \frac{\partial f_4}{\partial AB} & \frac{\partial f_4}{\partial Ab} & \frac{\partial f_4}{\partial ab} & \frac{\partial f_4}{\partial A_S B_S} & \frac{\partial f_4}{\partial A_S B} & \frac{\partial f_4}{\partial A_S b} \\ \frac{\partial f_5}{\partial AB} & \frac{\partial f_5}{\partial Ab} & \frac{\partial f_5}{\partial ab} & \frac{\partial f_5}{\partial A_S B_S} & \frac{\partial f_5}{\partial A_S B} & \frac{\partial f_5}{\partial A_S b} \\ \frac{\partial f_6}{\partial AB} & \frac{\partial f_6}{\partial Ab} & \frac{\partial f_6}{\partial ab} & \frac{\partial f_6}{\partial A_S B_S} & \frac{\partial f_6}{\partial A_S B} & \frac{\partial f_6}{\partial A_S b} \end{pmatrix}, \tag{14}$$

where partial derivatives are obtained via the quotient rule and are of the form

$$\frac{\partial f_1}{\partial AB} = \left(\frac{\partial g_1}{\partial AB} - g_1\frac{\partial \Omega}{\partial AB}\right)/\Omega^2, \qquad \frac{\partial f_1}{\partial Ab} = \left(\frac{\partial g_1}{\partial Ab} - g_1\frac{\partial \Omega}{\partial Ab}\right)/\Omega^2,$$
$$\frac{\partial f_1}{\partial ab} = \left(\frac{\partial g_1}{\partial ab} - g_1\frac{\partial \Omega}{\partial ab}\right)/\Omega^2, \qquad \frac{\partial f_1}{\partial A_S B_S} = \left(\frac{\partial g_1}{\partial A_S B_S} - g_1\frac{\partial \Omega}{\partial A_S B_S}\right)/\Omega^2,$$

$$\frac{\partial f_1}{\partial A_S B} = \left(\frac{\partial g_1}{\partial A_S B} - g_1\frac{\partial \Omega}{\partial A_S B}\right)/\Omega^2, \qquad \frac{\partial f_1}{\partial A_S b} = \left(\frac{\partial g_1}{\partial A_S b} - g_1\frac{\partial \Omega}{\partial A_S b}\right)/\Omega^2,$$
$$\frac{\partial f_2}{\partial AB} = \left(\frac{\partial g_2}{\partial AB} - g_2\frac{\partial \Omega}{\partial AB}\right)/\Omega^2, \qquad \frac{\partial f_2}{\partial Ab} = \left(\frac{\partial g_2}{\partial Ab} - g_2\frac{\partial \Omega}{\partial Ab}\right)/\Omega^2,$$
$$\frac{\partial f_2}{\partial ab} = \left(\frac{\partial g_2}{\partial ab} - g_2\frac{\partial \Omega}{\partial ab}\right)/\Omega^2, \qquad \frac{\partial f_2}{\partial A_S B_S} = \left(\frac{\partial g_2}{\partial A_S B_S} - g_2\frac{\partial \Omega}{\partial A_S B_S}\right)/\Omega^2,$$
$$\frac{\partial f_2}{\partial A_S B} = \left(\frac{\partial g_2}{\partial A_S B} - g_2\frac{\partial \Omega}{\partial A_S B}\right)/\Omega^2, \qquad \frac{\partial f_2}{\partial A_S b} = \left(\frac{\partial g_2}{\partial A_S b} - g_2\frac{\partial \Omega}{\partial A_S b}\right)/\Omega^2,$$
$$\frac{\partial f_3}{\partial AB} = \left(\frac{\partial g_3}{\partial AB} - g_3\frac{\partial \Omega}{\partial AB}\right)/\Omega^2, \qquad \frac{\partial f_3}{\partial Ab} = \left(\frac{\partial g_3}{\partial Ab} - g_3\frac{\partial \Omega}{\partial Ab}\right)/\Omega^2,$$
$$\frac{\partial f_3}{\partial ab} = \left(\frac{\partial g_3}{\partial ab} - g_3\frac{\partial \Omega}{\partial ab}\right)/\Omega^2, \qquad \frac{\partial f_3}{\partial A_S B_S} = \left(\frac{\partial g_3}{\partial A_S B_S} - g_3\frac{\partial \Omega}{\partial A_S B_S}\right)/\Omega^2,$$
$$\frac{\partial f_3}{\partial A_S B} = \left(\frac{\partial g_3}{\partial A_S B} - g_3\frac{\partial \Omega}{\partial A_S B}\right)/\Omega^2, \qquad \frac{\partial f_3}{\partial A_S b} = \left(\frac{\partial g_3}{\partial A_S b} - g_3\frac{\partial \Omega}{\partial A_S b}\right)/\Omega^2,$$
$$\frac{\partial f_4}{\partial AB} = \left(\frac{\partial g_4}{\partial AB} - g_4\frac{\partial \Omega}{\partial AB}\right)/\Omega^2, \qquad \frac{\partial f_4}{\partial Ab} = \left(\frac{\partial g_4}{\partial Ab} - g_4\frac{\partial \Omega}{\partial Ab}\right)/\Omega^2,$$
$$\frac{\partial f_4}{\partial ab} = \left(\frac{\partial g_4}{\partial ab} - g_4\frac{\partial \Omega}{\partial ab}\right)/\Omega^2, \qquad \frac{\partial f_4}{\partial A_S B_S} = \left(\frac{\partial g_4}{\partial A_S B_S} - g_4\frac{\partial \Omega}{\partial A_S B_S}\right)/\Omega^2,$$
$$\frac{\partial f_4}{\partial A_S B} = \left(\frac{\partial g_4}{\partial A_S B} - g_4\frac{\partial \Omega}{\partial A_S B}\right)/\Omega^2, \qquad \frac{\partial f_4}{\partial A_S b} = \left(\frac{\partial g_4}{\partial A_S b} - g_4\frac{\partial \Omega}{\partial A_S b}\right)/\Omega^2,$$
$$\frac{\partial f_5}{\partial AB} = \left(\frac{\partial g_5}{\partial AB} - g_5\frac{\partial \Omega}{\partial AB}\right)/\Omega^2, \qquad \frac{\partial f_5}{\partial Ab} = \left(\frac{\partial g_5}{\partial Ab} - g_5\frac{\partial \Omega}{\partial Ab}\right)/\Omega^2,$$
$$\frac{\partial f_5}{\partial ab} = \left(\frac{\partial g_5}{\partial ab} - g_5\frac{\partial \Omega}{\partial ab}\right)/\Omega^2, \qquad \frac{\partial f_5}{\partial A_S B_S} = \left(\frac{\partial g_5}{\partial A_S B_S} - g_5\frac{\partial \Omega}{\partial A_S B_S}\right)/\Omega^2,$$
$$\frac{\partial f_5}{\partial A_S B} = \left(\frac{\partial g_5}{\partial A_S B} - g_5\frac{\partial \Omega}{\partial A_S B}\right)/\Omega^2, \qquad \frac{\partial f_5}{\partial A_S b} = \left(\frac{\partial g_5}{\partial A_S b} - g_5\frac{\partial \Omega}{\partial A_S b}\right)/\Omega^2,$$
$$\frac{\partial f_6}{\partial AB} = \left(\frac{\partial g_6}{\partial AB} - g_6\frac{\partial \Omega}{\partial AB}\right)/\Omega^2, \qquad \frac{\partial f_6}{\partial Ab} = \left(\frac{\partial g_6}{\partial Ab} - g_6\frac{\partial \Omega}{\partial Ab}\right)/\Omega^2,$$
$$\frac{\partial f_6}{\partial ab} = \left(\frac{\partial g_6}{\partial ab} - g_6\frac{\partial \Omega}{\partial ab}\right)/\Omega^2, \qquad \frac{\partial f_6}{\partial A_S B_S} = \left(\frac{\partial g_6}{\partial A_S B_S} - g_6\frac{\partial \Omega}{\partial A_S B_S}\right)/\Omega^2,$$
$$\frac{\partial f_6}{\partial A_S B} = \left(\frac{\partial g_6}{\partial A_S B} - g_6\frac{\partial \Omega}{\partial A_S B}\right)/\Omega^2, \qquad \frac{\partial f_6}{\partial A_S b} = \left(\frac{\partial g_6}{\partial A_S b} - g_6\frac{\partial \Omega}{\partial A_S b}\right)/\Omega^2.$$

Within the above expressions, the partial derivative components are of the following form

$$
\begin{aligned}
\frac{\partial \Omega}{\partial AB} &= 2\varepsilon^4 AB + \varepsilon^3 \left(4\varepsilon_S A_S B + 4Ab\right) + \varepsilon^2 \left(2\varepsilon_S A_S B_S + 4\varepsilon_S A_S b + 2ab\right), \\
\frac{\partial \Omega}{\partial Ab} &= 4\varepsilon^3 AB + \varepsilon^2 \left(8\varepsilon_S A_S B + 4ab\right) + \varepsilon \left(4\varepsilon_S A_S B_S + 4\varepsilon_S A_S b\right), \\
\frac{\partial \Omega}{\partial ab} &= 2\varepsilon^2 AB + 4\varepsilon\varepsilon_S A_S B + 2\varepsilon_S^2 A_S B_S + 4\varepsilon_S A_S b + 2ab, \\
\frac{\partial \Omega}{\partial A_S B_S} &= 2\varepsilon^2 \varepsilon_S^2 AB + \varepsilon \left(4\varepsilon_S^3 A_S B + 4\varepsilon_S^2 Ab\right) + 2\varepsilon_S^4 A_S B_S + 4\varepsilon_S^3 A_S b + 2\varepsilon_S^2 ab, \\
\frac{\partial \Omega}{\partial A_S B} &= 4\varepsilon^3 \varepsilon_S AB + \varepsilon^2 \left(8\varepsilon_S^2 A_S B + 8\varepsilon_S Ab\right) + \varepsilon \left(4\varepsilon_S^3 A_S B_S + 8\varepsilon_S^2 A_S b + 4\varepsilon_S ab\right), \\
\frac{\partial \Omega}{\partial A_S b} &= 4\varepsilon^2 \varepsilon_S^2 AB + \varepsilon \left(8\varepsilon_S^2 A_S B + 4\varepsilon_S Ab\right) + 4\varepsilon_S^3 A_S B_S + 8\varepsilon_S^2 A_S b + 4\varepsilon_S ab, \\
\frac{\partial c}{\partial AB} &= 2\varepsilon^4 AB + 2\varepsilon^3 Ab + \frac{1}{2}\varepsilon^2 ab + \frac{1}{2}\varepsilon^2 \varepsilon_S^2 A_S B_S + 2\varepsilon^3 \varepsilon_S A_S B + \varepsilon^2 \varepsilon_S A_S b, \\
\frac{\partial c}{\partial Ab} &= 2\varepsilon^3 AB + \varepsilon^2 Ab + \varepsilon^2 \varepsilon_S A_S B, \\
\frac{\partial c}{\partial ab} &= \frac{1}{2}\varepsilon^2 AB, \\
\frac{\partial c}{\partial A_S B_S} &= \frac{1}{2}\varepsilon^2 \varepsilon_S^2 AB, \\
\frac{\partial c}{\partial A_S B} &= 2\varepsilon^3 \varepsilon_S AB + \varepsilon^2 \varepsilon_S Ab + \varepsilon^2 \varepsilon_S^2 A_S B, \\
\frac{\partial c}{\partial A_S b} &= \varepsilon^2 \varepsilon_S AB, \\
\frac{\partial d}{\partial AB} &= \varepsilon^3 Ab + \frac{1}{2}\varepsilon^2 ab + \frac{1}{2}\varepsilon^2 \varepsilon_S A_S b, \\
\frac{\partial d}{\partial Ab} &= \varepsilon^3 AB + \varepsilon^2 Ab + \frac{1}{2}\varepsilon\varepsilon_S^2 A_S B_S + \frac{3}{2}\varepsilon^2 \varepsilon_S A_S B + \frac{1}{2}\varepsilon\varepsilon_S A_S b, \\
\frac{\partial d}{\partial ab} &= \frac{1}{2}\varepsilon^2 AB + \frac{1}{2}\varepsilon\varepsilon_S A_S B, \\
\frac{\partial d}{\partial A_S B_S} &= \frac{1}{2}\varepsilon\varepsilon_S^2 Ab, \\
\frac{\partial d}{\partial A_S B} &= \frac{3}{2}\varepsilon^2 \varepsilon_S Ab + \frac{1}{2}\varepsilon\varepsilon_S ab + \frac{1}{2}\varepsilon\varepsilon_S^2 A_S b, \\
\frac{\partial d}{\partial A_S b} &= \frac{1}{2}\varepsilon^2 \varepsilon_S AB + \frac{1}{2}\varepsilon\varepsilon_S Ab + \frac{1}{2}\varepsilon\varepsilon_S^2 A_S B, \\
\frac{\partial e}{\partial AB} &= \frac{1}{2}\varepsilon^2 ab, \\
\frac{\partial e}{\partial Ab} &= \varepsilon^2 Ab + \varepsilon\varepsilon_S A_S b, \\
\frac{\partial e}{\partial ab} &= \frac{1}{2}\varepsilon^2 AB + 2ab + \frac{1}{2}\varepsilon_S^2 A_S B_S + \varepsilon\varepsilon_S A_S B + 2\varepsilon_S A_S b, \\
\frac{\partial e}{\partial A_S B_S} &= \frac{1}{2}\varepsilon\varepsilon_S^2 ab, \\
\frac{\partial e}{\partial A_S B} &= \varepsilon\varepsilon_S ab, \\
\frac{\partial e}{\partial A_S b} &= \varepsilon\varepsilon_S Ab + 2\varepsilon_S ab + \varepsilon_S^2 A_S b, \\
\frac{\partial f}{\partial AB} &= \frac{1}{2}\varepsilon^2 \varepsilon_S^2 A_S B_S, \\
\frac{\partial f}{\partial Ab} &= \varepsilon\varepsilon_S^2 A_S B_S, \\
\frac{\partial f}{\partial ab} &= \frac{1}{2}\varepsilon_S^2 A_S B_S,
\end{aligned}
$$

$$\frac{\partial f}{\partial A_S B_S} = \frac{1}{2}\varepsilon^2\varepsilon_S^2 AB + \varepsilon\varepsilon_S Ab + \frac{1}{2}\varepsilon_S^2 ab + 2\varepsilon_S^4 A_S B_S + 2\varepsilon\varepsilon_S^3 A_S B + 2\varepsilon^3 A_S b,$$
$$\frac{\partial f}{\partial A_S B} = 2\varepsilon\varepsilon_S^3 A_S B_S + \varepsilon^2\varepsilon_S^2 A_S B + \varepsilon\varepsilon_S^2 A_S b,$$
$$\frac{\partial f}{\partial A_S b} = 2\varepsilon_S^3 A_S B_S + \varepsilon\varepsilon_S^2 A_S B + \varepsilon_S^2 A_S b,$$
$$\frac{\partial g}{\partial AB} = \frac{1}{2}\varepsilon^2\varepsilon_S^2 A_S B_S + \varepsilon^3\varepsilon_S A_S B + \frac{1}{2}\varepsilon^2\varepsilon_S A_S b,$$
$$\frac{\partial g}{\partial Ab} = \frac{1}{2}\varepsilon^2\varepsilon_S A_S B + \frac{1}{2}\varepsilon\varepsilon_S^2 A_S B_S + \varepsilon^2\varepsilon_S A_S B + \frac{1}{2}\varepsilon\varepsilon_S A_S b,$$
$$\frac{\partial g}{\partial ab} = \frac{1}{2}\varepsilon\varepsilon_S A_S B,$$
$$\frac{\partial g}{\partial A_S B_S} = \frac{1}{2}\varepsilon^2\varepsilon_S^2 AB + \frac{1}{2}\varepsilon\varepsilon_S^2 Ab + \varepsilon\varepsilon_S^3 A_S B,$$
$$\frac{\partial g}{\partial A_S B} = \varepsilon^3\varepsilon_S AB + \frac{1}{2}\varepsilon^2\varepsilon_S Ab + \varepsilon^2\varepsilon_S Ab + \varepsilon\varepsilon_S^3 A_S B_S + 2\varepsilon^2\varepsilon_S^2 A_S B + \varepsilon^2\varepsilon_S^2 A_S B + \frac{1}{2}\varepsilon\varepsilon_S ab + \frac{3}{2}\varepsilon\varepsilon_S^2 A_S b,$$
$$\frac{\partial g}{\partial A_S b} = \frac{1}{2}\varepsilon^2\varepsilon_S AB + \frac{1}{2}\varepsilon\varepsilon_S Ab + \frac{3}{2}\varepsilon\varepsilon_S A_S B,$$
$$\frac{\partial h}{\partial AB} = \frac{1}{2}\varepsilon^2\varepsilon_S A_S b,$$
$$\frac{\partial h}{\partial Ab} = \frac{1}{2}\varepsilon\varepsilon_S^2 A_S B_S + \frac{1}{2}\varepsilon^2\varepsilon_S A_S B + \frac{1}{2}\varepsilon\varepsilon_S A_S b,$$
$$\frac{\partial h}{\partial ab} = \frac{1}{2}\varepsilon_S^2 A_S B_S + \varepsilon_S A_S b,$$
$$\frac{\partial h}{\partial A_S B_S} = \frac{1}{2}\varepsilon\varepsilon_S^2 Ab + \frac{1}{2}\varepsilon_S^2 ab + \frac{1}{2}\varepsilon\varepsilon_S A_S B + \varepsilon_S^3 A_S b,$$
$$\frac{\partial h}{\partial A_S B} = \frac{1}{2}\varepsilon^2\varepsilon_S Ab + \frac{1}{2}\varepsilon\varepsilon_S ab + \frac{3}{2}\varepsilon\varepsilon_S^2 A_S b,$$
$$\frac{\partial h}{\partial A_S b} = \frac{1}{2}\varepsilon^2\varepsilon_S AB + \frac{1}{2}\varepsilon\varepsilon_S Ab + \varepsilon_S ab + \varepsilon_S^2 A_S B_S + \frac{3}{2}\varepsilon\varepsilon_S^2 A_S B + 2\varepsilon_S^2 A_S b + \varepsilon_S^2 A_S b.$$

The eigenvalues of a given equilibrium state are then obtained by substituting equilibrium states for each variable into the Jacobian matrix and solving

$$\det|J - \lambda I| = 0, \tag{15}$$

where, as before, λ denotes an eigenvalue and I the identity matrix with dimensions equal to those of J.

We now solve equation (15) to explore whether the stability properties of either biologically feasible equilibrium state (i.e. transgene elimination or transgene introgression through releases into the environment) are altered by the inclusion of free-suppressor constructs into the model. As before we find that the transgene elimination (or equivalently the no transgenic release) equilibrium is stable for any combination of transgene and free-suppressor relative fitness parameters, assuming they do not confer a fitness advantage over wild-type (i.e. $\varepsilon \leq 1$ and $\varepsilon_S \leq 1$). We do however, observe a difference in the stability properties of the transgene introgression equilibrium. In particular, we find that the introduction of free-suppressor constructs into this model allows this equilibrium state to become unstable for certain combinations of relative fitness parameters ε and ε_S. Eigenvalues obtained from both the transgene elimination and transgene introgression equilibria are shown in Figure 2 for some illustrative ε and ε_S parameter values.

Since the example in Figure 2(e) shows that the transgene introgression equilibrium can become unstable in cases where $\varepsilon = \varepsilon_S$, we now investigate whether this equilibrium can become unstable (i.e. reversible by introduction of free-suppressor constructs) for parameter combinations whereby the free-suppressor constructs confer a fitness disadvantage relative to the initially introduced transgenic constructs ($\varepsilon_S < \varepsilon$). To investigate this we perform repeated numerical simulations of the model in equations (7)-(12) with a given value of ε and gradually increasing values of ε_S. This allows us to find the smallest ε_S parameter that enables reversal to a wild-type population for a given ε. We repeat this for different sizes of introductions for free-suppressor carrying individuals. In particular, we consider the release of free suppressor carrying individuals to occur 500 generations after the initial transgenic introduction so as to allow that system to

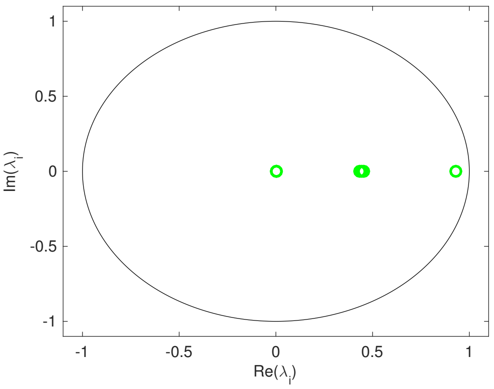

(a) Transgene Elimination ($ab = 1$)
$\varepsilon = 0.95$, $\varepsilon_S = 0.93$

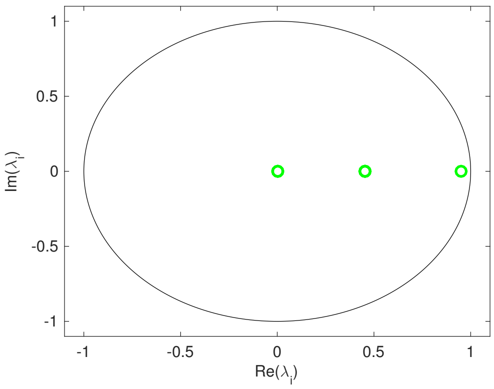

(b) Transgene Elimination ($ab = 1$)
$\varepsilon = 0.95$, $\varepsilon_S = 0.95$

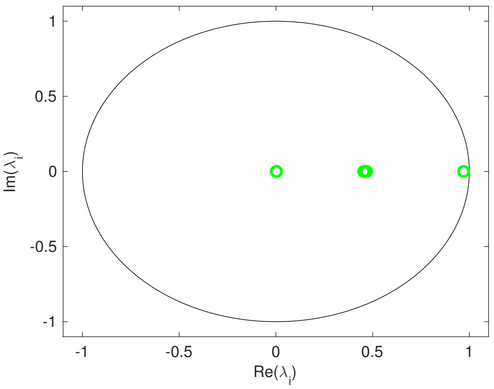

(c) Transgene Elimination ($ab = 1$)
$\varepsilon = 0.95$, $\varepsilon_S = 0.97$

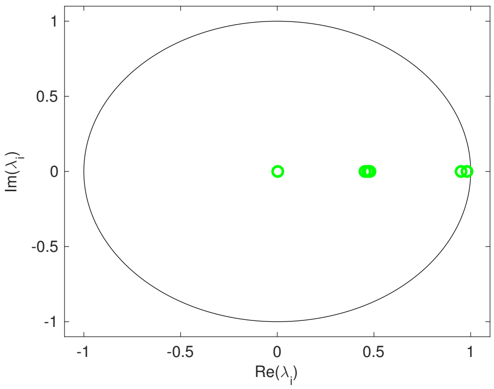

(d) Transgene Introgression ($0 < ab, Ab, AB < 1$)
$\varepsilon = 0.95$, $\varepsilon_S = 0.93$

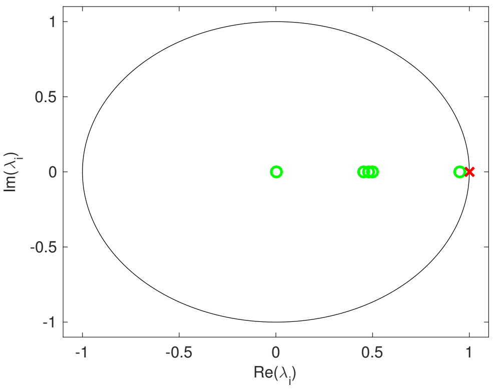

(e) Transgene Introgression ($0 < ab, Ab, AB < 1$)
$\varepsilon = 0.95$, $\varepsilon_S = 0.95$

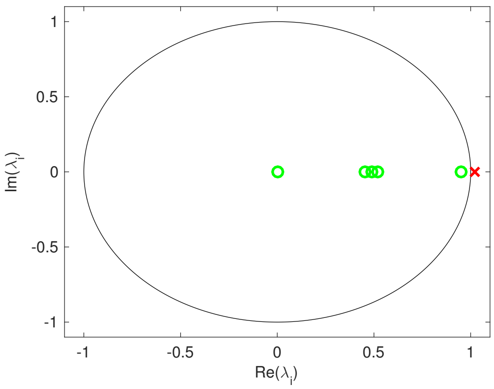

(f) Transgene Introgression ($0 < ab, Ab, AB < 1$)
$\varepsilon = 0.95$, $\varepsilon_S = 0.97$

Figure 2: **Eigenvalues of various equilibrium states in a model including free-suppressor constructs.** Here green circles represent eigenvalues with absolute value less than one and red crosses eigenvalues with absolute value greater than one. The top row of plots (i.e. (a)-(c)) show eigenvalues associated with an equilibrium where all transgenes have been eliminated. These show that for some example combinations of relative fitness parameters with $\varepsilon > \varepsilon_S$, $\varepsilon = \varepsilon_S$ and $\varepsilon < \varepsilon_S$ (details in panel labels) this equilibrium is stable. The bottom row (i.e. (d)-(f)) show eigenvalues associated with a transgene introgression equilibrium (achieved through releases of transgenic individuals into the environment) for the same relative fitness parameter combinations used in (a)-(c). For these parameters the case where $\varepsilon > \varepsilon_S$ is stable whereas cases where $\varepsilon \leq \varepsilon_S$ the equilibrium is unstable, suggesting reversal through free-suppressor introductions is feasible.

reach the introgression equilibrium using the following conditions

$$AB_{t+1} = \Big[\varepsilon^4 AB_t^2 + 2\varepsilon^3 AB_t Ab_t + \frac{1}{2}\varepsilon^2 AB_t ab_t + \frac{1}{2}\varepsilon^2 Ab_t^2 + \frac{1}{2}\varepsilon^2\varepsilon_S^2 AB_t A_S B_{S_t} + 2\varepsilon^3\varepsilon_S AB_t A_S B_t \qquad (16)$$
$$+\varepsilon^2\varepsilon_S AB_t A_S b_t + \varepsilon^2\varepsilon_S Ab_t A_S B_t + \frac{1}{2}\varepsilon^2\varepsilon_S^2 A_S B_t^2\Big] /\Omega_t(1+\alpha_S),$$

$$Ab_{t+1} = \Big[\varepsilon^3 AB_t Ab_t + \frac{1}{2}\varepsilon^2 AB_t ab_t + \frac{1}{2}\varepsilon^2\varepsilon_S AB_t A_S b_t + \frac{1}{2}\varepsilon^2 Ab_t^2 + \frac{1}{2}\varepsilon\varepsilon_S^2 Ab_t A_S B_{S_t} \qquad (17)$$
$$+\frac{3}{2}\varepsilon^2\varepsilon_S Ab_t A_S B_t + \frac{1}{2}\varepsilon\varepsilon_S Ab_t A_S b_t + \frac{1}{2}\varepsilon\varepsilon_S ab_t A_S B_t + \frac{1}{2}\varepsilon\varepsilon_S^2 A_S B_t A_S b_t\Big] /\Omega_t(1+\alpha_S),$$

$$ab_{t+1} = \Big[\frac{1}{2}\varepsilon^2 AB_t ab_t + \frac{1}{2}\varepsilon^2 Ab_t^2 + \varepsilon\varepsilon_S Ab_t A_S b_t + ab_t^2 + \frac{1}{2}\varepsilon_S^2 ab_t A_S B_S + \varepsilon\varepsilon_S ab_t A_S B_t \qquad (18)$$
$$+2\varepsilon_S ab_t A_S b_t + \frac{1}{2}\varepsilon_S^2 A_S b^2\Big] /\Omega_t(1+\alpha_S),$$

$$A_S B_{S_{t+1}} = \Big[\frac{1}{2}\varepsilon^2\varepsilon_S^2 AB_t A_S B_{S_t} + \varepsilon\varepsilon_S^2 Ab_t A_S B_{S_t} + \frac{1}{2}\varepsilon_S^2 ab_t A_S B_{S_t} + \varepsilon_S^4 A_S B_{S_t}^2 + 2\varepsilon\varepsilon_S^3 A_S B_{S_t} A_S B_t \qquad (19)$$
$$+2\varepsilon_S^3 A_S B_{S_t} A_S b_t + \frac{1}{2}\varepsilon^2\varepsilon_S^2 A_S B_t^2 + \varepsilon\varepsilon_S^2 A_S B_t A_S b_t + \frac{1}{2}\varepsilon_S A_S b_t^2 + \alpha_S\Omega_t\Big] /\Omega_t(1+\alpha_S),$$

$$A_S B_{t+1} = \Big[\frac{1}{2}\varepsilon^2\varepsilon_S^2 AB_t A_S B_{S_t} + \varepsilon^3\varepsilon_S AB_t A_S B_t + \frac{1}{2}\varepsilon^2\varepsilon_S AB_t A_S b_t + \frac{1}{2}\varepsilon^2\varepsilon_S Ab_t A_S B_t \qquad (20)$$
$$+\frac{1}{2}\varepsilon\varepsilon_S^2 Ab_t A_S B_{S_t} + \varepsilon^2\varepsilon_S Ab_t A_S B_t + \frac{1}{2}\varepsilon\varepsilon_S Ab_t A_S b_t + \varepsilon\varepsilon_S^3 A_S B_{S_t} + \frac{3}{2}\varepsilon^2\varepsilon_S^2 A_S B_t^2$$
$$+\frac{3}{2}\varepsilon\varepsilon_S^2 A_S B_t A_S b_t + \frac{1}{2}\varepsilon\varepsilon_S ab_t A_S B_t\Big] /\Omega_t(1+\alpha_S),$$

$$A_S b_{t+1} = \Big[\frac{1}{2}\varepsilon^2\varepsilon_S AB_t A_S b_t + \frac{1}{2}\varepsilon\varepsilon_S AB_t A_S B_{S_t} + \frac{1}{2}\varepsilon^2\varepsilon_S^2 Ab_t A_S B_t + \frac{1}{2}\varepsilon\varepsilon_S Ab_t A_S b_t \qquad (21)$$
$$+\frac{1}{2}\varepsilon_S^2 ab_t A_S B_{S_t} + \frac{1}{2}\varepsilon\varepsilon_S ab_t A_S B_t + \varepsilon_S ab_t A_S b_t + \varepsilon_S^3 A_S B_{S_t} A_S b_t + \frac{3}{2}\varepsilon\varepsilon_S A_S B A_S b$$
$$+\varepsilon_S A_S b_t^2 + \frac{1}{2}\varepsilon_S^2 A_S b_t^2\Big] /\Omega_t(1+\alpha_S),$$

where $t = 499$ and α_S denotes the release ratio of free-suppressor carrying individuals. This release ratio is calculated as α_S=introduced/wild of all genotypes and this release is assumed to be of individuals homozygous for both free-suppressor constructs (i.e. we only consider $A_S B_S$ haplotype introductions). Results of this investigation are shown in Figure 3 for three different free-suppressor release ratios, namely α_S =0.1, 1 and 10.

These results clearly show that reversal of an initial transgenic release is feasible using releases of free-suppressor carrying individuals so long as those free-suppressor constructs do not confer too great of a fitness deficit relative to the initially released transgenic constructs. As can be seen in the results of Figure 3, the fitness deficit that can be tolerated is larger for greater free-suppressor release ratios and also for initially released transgenic constructs with lower relative fitness.

To confirm the results of Figure 3 and explore this behaviour further we numerically simulate this system for a range of initial transgenic construct and free-suppressor relative fitness parameters and extract various indicators of the observed behaviour. In particular, we examine the final frequency of the initially introduced transgenes, wild-type alleles and free-suppressor constructs, the maximum frequency attained by the free-suppressors and the time taken to return the system to wild-type (measured as the time from the release of free-suppressors until wild-type alleles return to a frequency greater than 0.95). For free-suppressor release ratios of α_S =0.1, 1 and 10, allele frequency results are are shown in Figures 4, 5 and 6, respectively and time to reversal results are given in Figure 7.

These results support those from Figure 3 in suggesting that free-suppressor constructs that confer a fitness deficit relative to the initially introduced constructs can lead to a reversal of the two-locus underdominance system and thus a return to a fully wild-type population so long as that deficit is not too large. In these results the left hand portion of each plot represents the case where ε is too low for

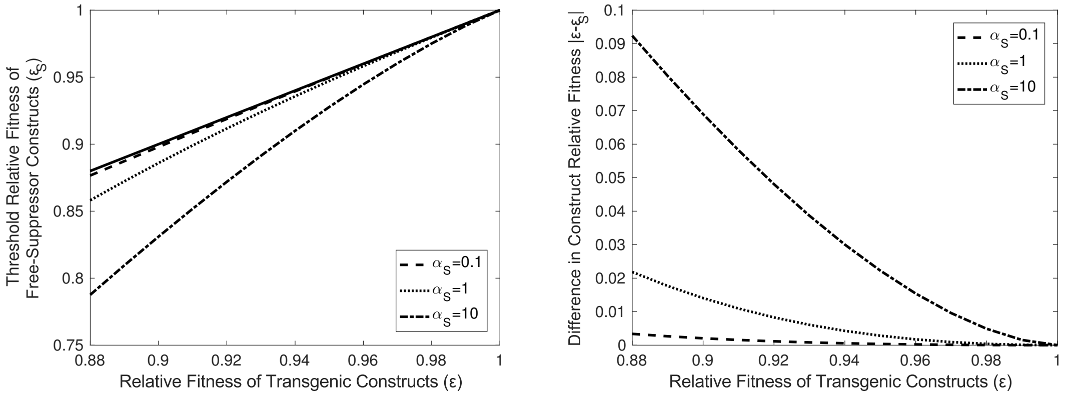

(a) Threshold Relative Fitness for Transgene Elimination

(b) Relative Fitness Differences Allowing Transgene Elimination

Figure 3: **Free-suppressor transgenes with lower relative fitness than the initially introduced transgenes can give reversal to a wild-type population.** Panel (a) shows, for a given free-suppressor release ratio, the free-suppressor relative fitness (ε_S) that can give a return to a wild-type population where the initially introduced transgenic constructs are of relative fitness ε. Panel (b) shows the tolerable difference between the relative fitness of free-suppressor and initial transgenic constructs for three given free-suppressor release ratios. This is essentially the difference between the threshold lines and the solid line from panel (a).

the initial system to achieve introgression from a 1:1 (introduced:wild) release. The lower-right regions represent the non-reversed transgene introgression equilibrium, whereas the upper-right region represents the region in which reversal was achieved. Aside from the size of tolerable fitness difference between transgenic and free-suppressor constructs, the main difference seen between results here is in the maximum frequency attained by free-suppressor alleles for different free-suppressor release ratios. In particular we see that larger introductions of free-suppressor carriers leads to higher maximum frequencies for the free-suppressor allele. However, Figure 7 shows that in spite of the notable differences in maximum free-suppressor frequency, the number of generations taken to return to wild-type is not drastically altered. This is likely due to a balancing between the longer time taken for small releases to rise to their maximum frequency and the longer time for larger releases that attain higher frequencies to fall and be eliminated.

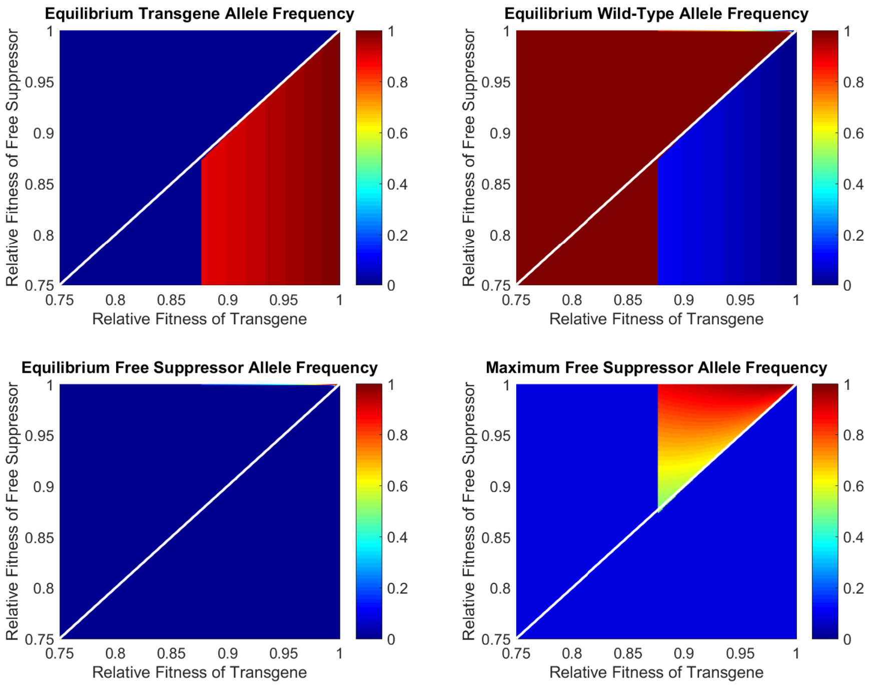

Figure 4: **Equilibrium and maximum allele frequencies resulting from an initial 1:1 (introduced:wild) UD release and a free-suppressor release at ratio $\alpha_S = 0.1$ made five hundred generations later.** Here colours relate to the relevant equilibrium/maximum frequencies attained for a given pair of ε and ε_S parameters. White diagonals simply represent the line whereby $\varepsilon = \varepsilon_S$.

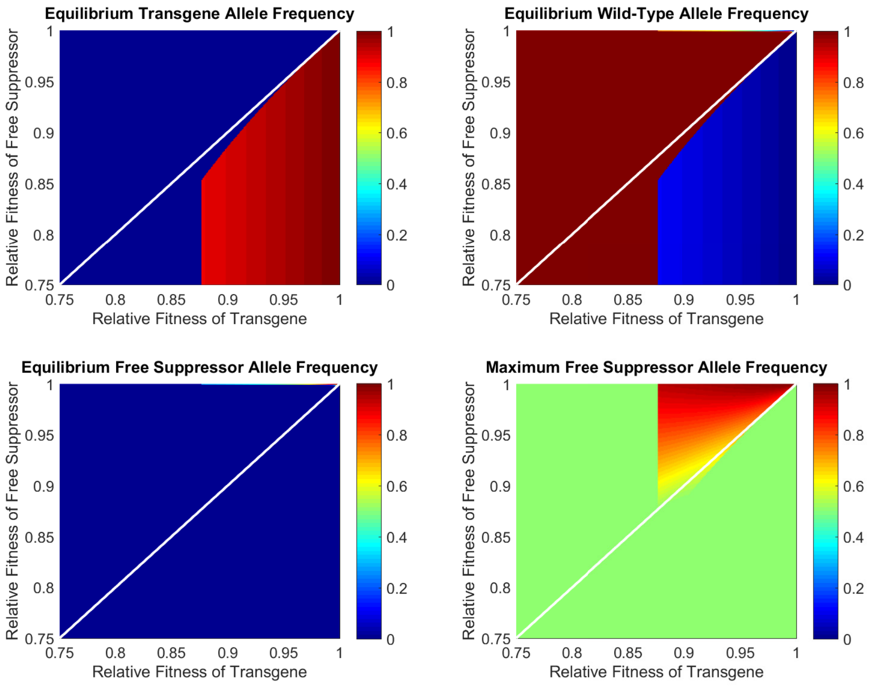

Figure 5: **Equilibrium and maximum allele frequencies resulting from an initial 1:1 (introduced:wild) UD release and a free-suppressor release at ratio $\alpha_S = 1$ made five hundred generations later.** Here colours relate to the relevant equilibrium/maximum frequencies attained for a given pair of ε and ε_S parameters. White diagonals simply represent the line whereby $\varepsilon = \varepsilon_S$.

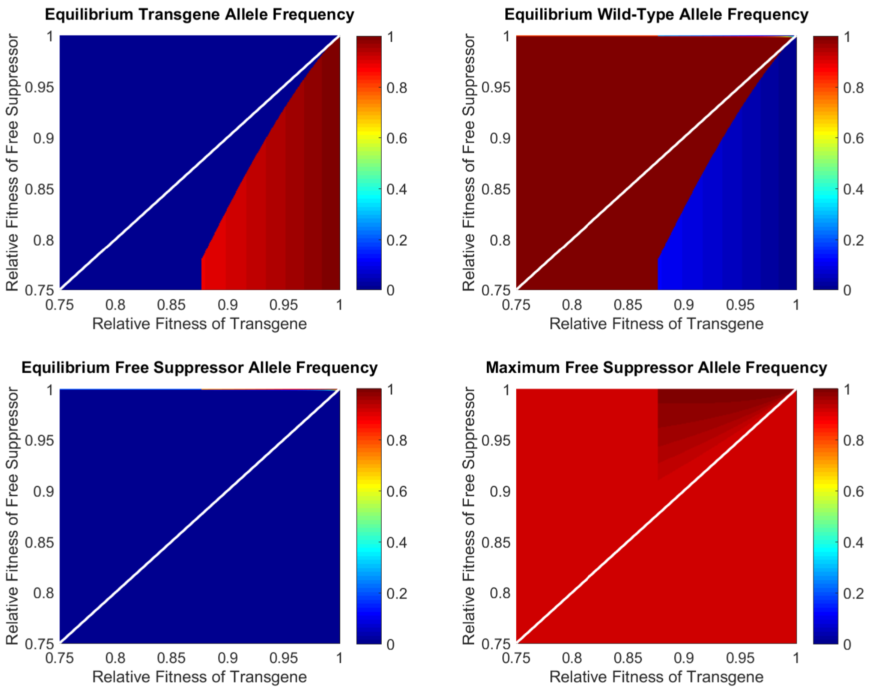

Figure 6: **Equilibrium and maximum allele frequencies resulting from an initial 1:1 (introduced:wild) UD release and a free-suppressor release at ratio $\alpha_S = 10$ made five hundred generations later.** Here colours relate to the relevant equilibrium/maximum frequencies attained for a given pair of ε and ε_S parameters. White diagonals simply represent the line whereby $\varepsilon = \varepsilon_S$.

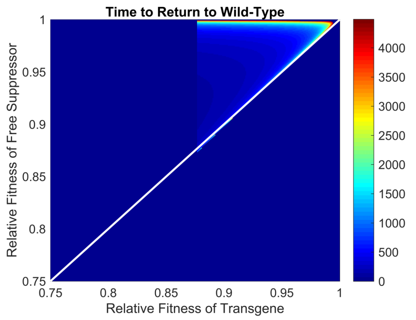

(a) $\alpha_S = 0.1$

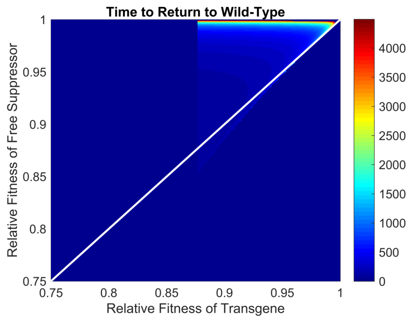

(b) $\alpha_S = 1$

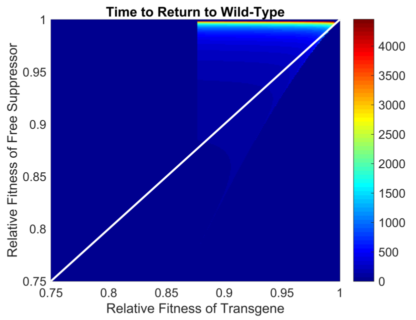

(c) $\alpha_S = 10$

Figure 7: **Time taken for an introduction of free-suppressor carriers to return a wild-type population.** Here colours the number of generations from time of release until a return to wild-type. Where the colour indicates zero generations this means either that the initial system did not achieve introgression or the free-suppressors failed to return a wild-type population. White diagonals simply represent the line whereby $\varepsilon = \varepsilon_S$.
